# Heterotopic pregnancy: a case report about triplets

**DOI:** 10.1093/bjrcr/uaaf040

**Published:** 2025-07-28

**Authors:** Sara Cherkaoui, Lina Belkouchi, Badra Idrissi, Siham EL HADDAD, Nazik Allali, Latifa Chat

**Affiliations:** Department of Radiology, Children Hospital of Rabat, Ibn Sina University Hospital, Faculty of medicine and pharmacy of Rabat, University Mohammed V, Rabat, 10100, Morocco; Department of Radiology, Children Hospital of Rabat, Ibn Sina University Hospital, Faculty of medicine and pharmacy of Rabat, University Mohammed V, Rabat, 10100, Morocco; Department of Gynecology, Children Hospital of Rabat, Ibn Sina University Hospital, Faculty of medicine and pharmacy of Rabat, University Mohammed V, Rabat, 10100, Morocco; Department of Radiology, Children Hospital of Rabat, Ibn Sina University Hospital, Faculty of medicine and pharmacy of Rabat, University Mohammed V, Rabat, 10100, Morocco; Department of Radiology, Children Hospital of Rabat, Ibn Sina University Hospital, Faculty of medicine and pharmacy of Rabat, University Mohammed V, Rabat, 10100, Morocco; Department of Radiology, Children Hospital of Rabat, Ibn Sina University Hospital, Faculty of medicine and pharmacy of Rabat, University Mohammed V, Rabat, 10100, Morocco

**Keywords:** heterotopic pregnancy, triplets, pelvic ultrasound, MRI, case report

## Abstract

Heterotopic pregnancy refers to the concomitant presence of an intrauterine pregnancy and an ectopic pregnancy (EP). It is rare and more frequently found in women who have undergone medically assisted procreation. An abdominal location of the ectopic gestational sac is even less common, accounting for 1.4% of all ectopic pregnancies, conferring a high risk of morbidity and mortality. Diagnosis can sometimes be difficult, and pelvic ultrasound alone may not be sufficient. Additional pelvic MRI may be useful to confirm the diagnosis with certainty. Heterotopic pregnancy requires urgent management and is mainly treated surgically, although there are certain situations where medical treatment may be indicated. We report the case of a 29-year-old patient admitted with pelvic pain and mild metrorrhagia, with an elevated beta-hCG level suggesting the diagnosis of an EP, which was confirmed by ultrasound and MRI, revealing the presence of 3 foetuses: 1 was developing in the abdominal cavity while the other 2 were normally present in the uterine cavity.

## Introduction

Heterotopic pregnancy is a rare form of ectopic pregnancy (EP). It represents a real diagnostic challenge because of the ambiguous clinical signs, the elevated beta-human chorionic gonadotropin (hCG) levels, and the visualization of an intrauterine pregnancy (IUP), which can be falsely reassuring and delay the diagnosis until complications arise (rupture), especially if the woman has undergone medically assisted procreation (MAP). When the EP is located in the abdomen, it may progress beyond the first trimester, increasing the risk of rupture of the ectopic sac, possibly threatening the mother’s vital prognosis.

Pelvic ultrasound is the modality of choice. It is used to look for an extra-uterine gestational sac along or outside the internal genital tract, associated with an intra-uterine pregnancy whose evolutivity and viability must be carefully appreciated. A thorough knowledge of the ultrasound signs enables the diagnosis to be made at an early stage and emergency treatment to be provided, usually by surgery. In cases of diagnostic uncertainty, additional pelvic MRI in advanced gestational age pregnancies can help determine the appropriate diagnosis, thus avoiding invasive explorative surgical procedures.

## Clinical presentation

The patient was a 29-year-old woman with no medical history records, nulliparous, who had undergone hormonal stimulation, that is, ovarian induction by medical treatment, in order to get pregnant. She presented with pelvic pain and mild metrorrhagia that had been evolving for approximately 1 month. These symptoms bore no resemblance to the patient’s usual menstrual periods, and there were no signs of infection. Furthermore, the patient was approximately 13 weeks overdue for her normal menstrual period and was unaware of her pregnancy. As the pelvic pain had become more intense, the patient decided to go to the emergency department, where a beta-HCG test was done and revealed markedly elevated levels >225,000.00 mIU/ml.

## Investigations/imaging findings

**Figure 1.**
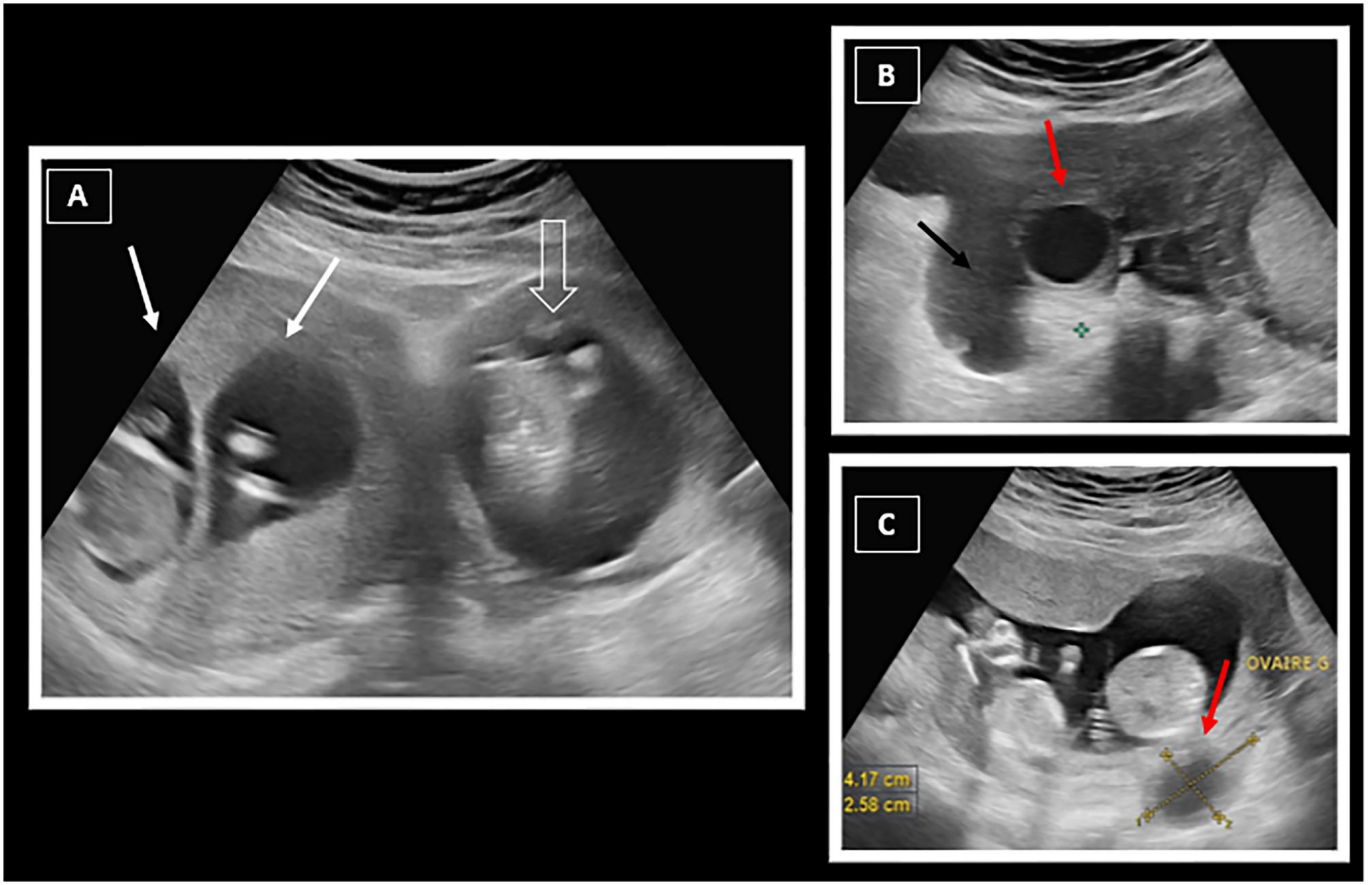
Trans-abdominal ultrasound images obtained using a low-frequency convex probe showing an evolving triplet pregnancy. (A) Two gestational sacs are seen in the uterine cavity (white arrows), while a third adjacent gestational sac is lateralized to the left, but does not appear to be developing within the uterus (empty arrow). (B and C) Enlarged and follicular right (B) and left (C) ovaries are visualized on either side of the uterus, probably in relation to ovarian hyperstimulation syndrome (red arrows). Note the finely echogenic peritoneal effusion in (B), suggesting haemoperitoneum (black arrow).

On clinical examination, the patient was haemodynamically stable with a normal blood pressure (BP = 122/62) and a normal heart rate (HR = 94bpm). Palpation revealed tenderness in the left iliac fossa. Her blood test showed normocytic normochromic anaemia with a low haemoglobin level of 6 g/dl. Given the suspicion of an EP, a pelvic ultrasound was performed. It revealed a bi-foetal, bi-chorionic, and bi-amniotic IUP, as well as a third gestational sac lateralized to the left, which appeared to be outside the uterine cavity, containing a foetus with an estimated gestational age of 13 weeks, whose placenta was separate from the others. The pregnancy was progressing well, with 3 foetuses having positive cardiac activity and good morphological development. The ultrasound scan also revealed enlarged, multi-follicular ovaries, suggesting ovarian hyperstimulation syndrome, as well as a finely echogenic intraperitoneal effusion at the pouch of Douglas and in the left paracolic gutter, probably related to haemoperitoneum (Figure 1).

Overall, ultrasound revealed an intrauterine gemellar pregnancy and a probable left latero-uterine EP associated with moderate haemoperitoneum and ovarian hyperstimulation syndrome.

An additional MRI was performed in an emergency setting, using a Siemens 1.5 Tesla machine, with a phased-array surface body coil, to confirm the diagnosis of heterotopic pregnancy and to specify its location.

**Figure 2.**
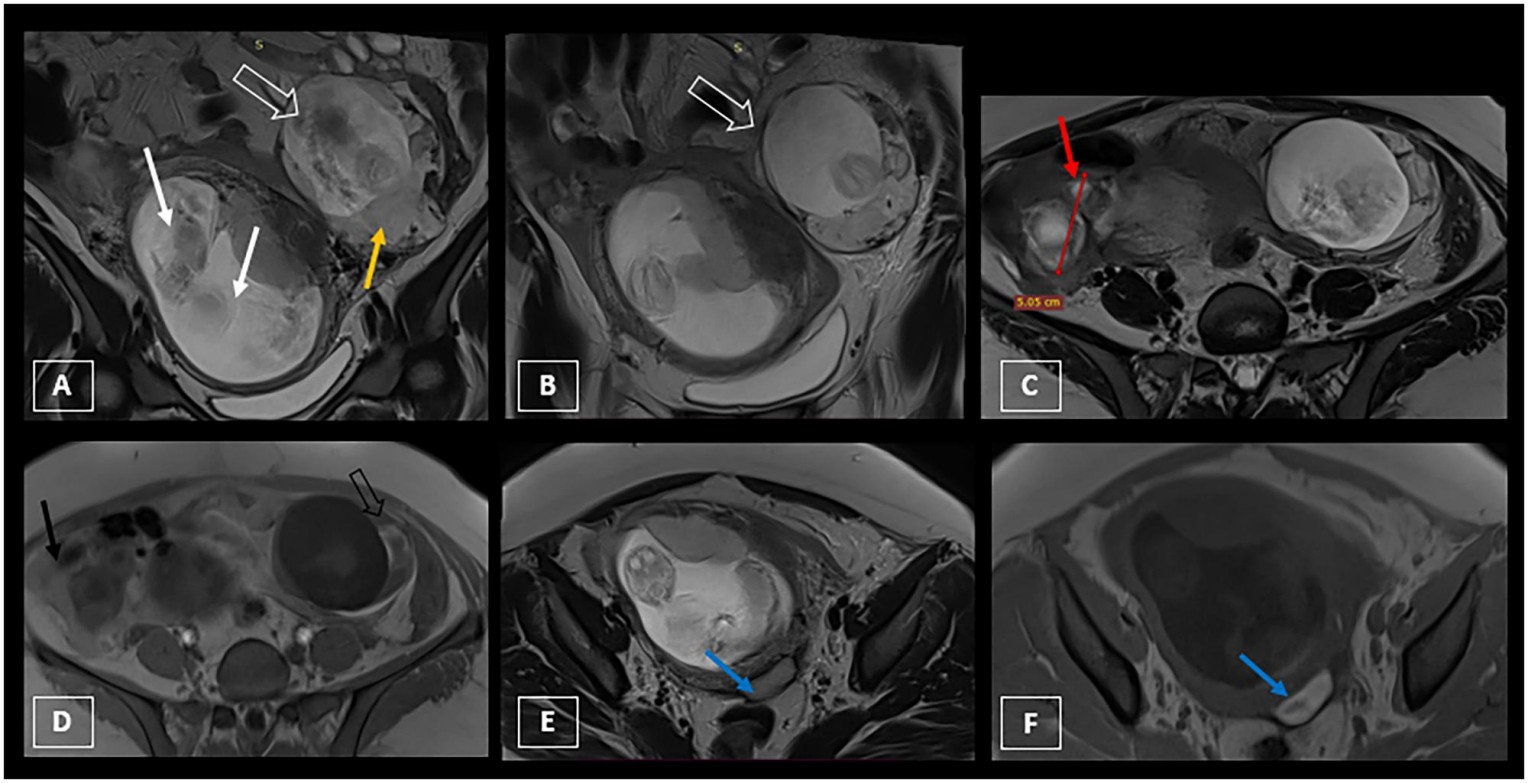
Pelvic MRI showing a heterotopic triplet pregnancy. (A and B) Coronal T2-weighted images showing a bifoetal, bichorionic, and bi amniotic intrauterine pregnancy (white arrows) associated with a third gestational sac lateralized to the left, located intra-abdominally (empty white arrow), not surrounded by myometrium, with its own placenta (yellow arrow). (C) T2-weighted axial image showing the free ectopic sac in the abdominal cavity. Note the right ovary that is enlarged and multi-follicular (red arrow), consistent with ovarian hyperstimulation syndrome. (D) T1-weighted axial section showing blood around the ectopic placenta (black empty arrow) and haemoperitoneum (black arrow), suggesting signs of early rupture of the ectopic gestational sac. (E) T2-weighted axial section and (F) T1-weighted axial section showing an associated left haematosalpinx (blue arrow) that is probably due to an undetected rupture of the fallopian tube, which resulted in the extrusion of the zygote into the peritoneal cavity, suggesting a secondary origin of the abdominal heterotopic pregnancy.

The protocol included Haste and TruFi T2-weighted sequences in the axial, coronal, and sagittal planes, as well as an axial T1 sequence, both with and without fat saturation, without injection of contrast medium. The MRI clearly showed the third gestational sac in the abdominal cavity adjacent to the uterus, not surrounded by myometrium, containing a foetus and placenta of its own. It also confirmed the haemoperitoneum as the peritoneal fluid showed hyperintensity on T1-weighted images, with signs indicating the beginning of rupture of the ectopic gestational sac (Figure 2).

## Differential diagnosis

A markedly elevated beta-HCG levels may simply indicate the presence of a normal IUP, inevitably a twin or triplet pregnancy. However, when it is associated with abdominal pain, the main concern should be to determine whether there is an EP, which is a real diagnostic and therapeutic emergency. Pelvic ultrasound is the preferred method of examination to help guide the patient’s management.

This clinical presentation, along with the echographic findings, may also suggest a twin pregnancy evolving within a uterine malformation, in particular a bicornuate uterus, making it important to perform a pelvic MRI whenever there is the slightest doubt. Fortunately for us, the patient had already undergone pelvic imaging techniques as part of her pre-treatment work-up, ruling out uterine malformation.

## Treatment

**Figure 3.**
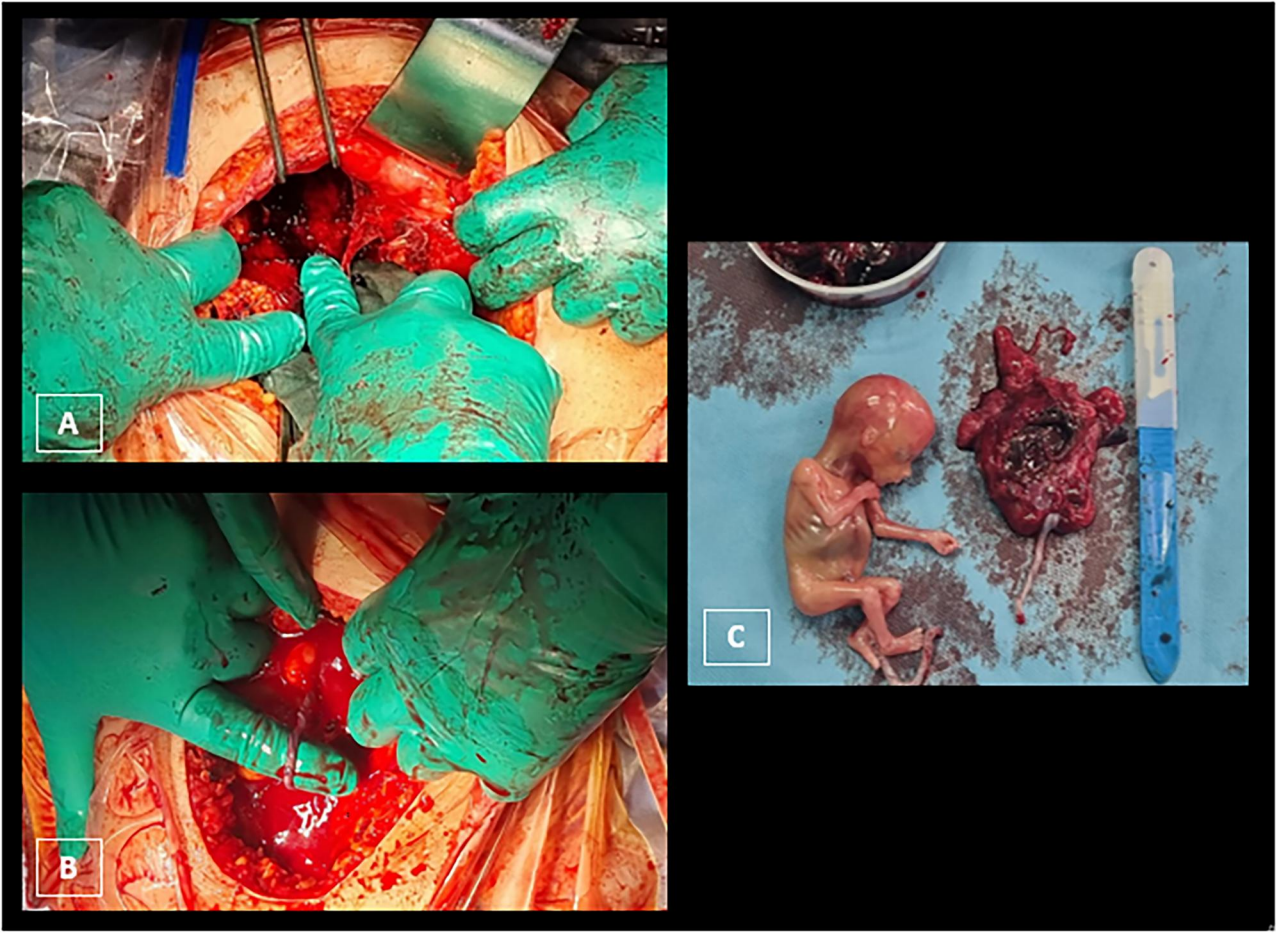
Intra-operative laparotomy images. A and B reveal the presence of a foetus within the abdominal cavity with an associated haemorrhagic effusion. (C) Real size of the ectopic foetus and his placenta.

The patient was immediately transferred to the operating room for surgical intervention, which was performed via laparotomy using a Pfannenstiel incision, where the third intra-abdominal gestational sac was successfully removed. This approach was chosen in view of the haemoperitoneum that was observed on radiological examinations. The sac was indeed ruptured, but the adjacent intestinal loops were able to seal the breach (Figure 3). The 2 intra-uterine gestational sacs were not affected by the surgical procedure.

## Outcome, follow-up, and discussion

The patient was discharged a few days later with good clinical evolution of her intra-uterine twin pregnancy. Follow-up was conducted until full-term vaginal delivery without complications.

Heterotopic pregnancy is defined as the simultaneous presence of an IUP and an extra uterine pregnancy (EUP). It is more frequently found in patients who have undergone MAP, ranging from ovarian induction by medical treatment to in vitro fertilization (IVF).^1^ The incidence is much lower in women conceiving naturally, who are more prone to an isolated EP.^2^

Risk factors are similar to those of EUP, including: advanced maternal age, endometriosis, pelvic inflammatory disease, voluntary or therapeutic termination of pregnancy, a history of EP and abdominal or pelvic surgery.^3^

Diagnosing a heterotopic pregnancy can be challenging and often delayed due to less pronounced symptoms. An existing IUP identified on imaging, combined with elevated beta-hCG levels, can obscure a concurrent EP, especially when an ectopic sac is not clearly visualized early in the pregnancy. Sometimes, the diagnosis is only made when the ectopic gestational sac ruptures. This highlights the importance of checking the adnexa, cervix, and the entire abdominal cavity during the first-trimester obstetric ultrasound scan, especially in the case of a woman who has undergone MAP.^2^

Pelvis ultrasound (US), using both transabdominal and transvaginal approaches, is the modality of choice and gold standard for assessing EP.^4^ It is used to look for echographic signs of an extra-uterine gestational sac, depending on its location, with tubal location being the most frequent, associated with an intra-uterine pregnancy whose evolutivity and viability must be ensured. In cases of abdominal ectopic gestation, there is a considerable morbidity and mortality risk for the mother and foetus due to the risk of massive blood loss from incomplete or entire placental separation. The trophoblast may also invade the maternal abdominal organs, potentially causing heavy bleeding or organ rupture. In US, abdominal pregnancy is diagnosed by identifying a gestational sac with surrounding thick echogenic margins that is implanted within the peritoneal cavity. The use of Doppler US can facilitate the location of the gestational sac among the surrounding loops of bowel by identifying peritrophoblastic flow around the sac. The presence of echogenic free fluid suggests hemoperitoneum. Ovaries may appear enlarged due to ovarian hyperstimulation, thereby making it challenging to visualize the EP.^5^

Ultrasound diagnosis can sometimes be difficult due to the presence of a falsely reassuring intrauterine gestational sac in a patient who is initially mildly symptomatic or asymptomatic, with or without ovarian hyperstimulation syndrome.^6^

Intra-abdominal location is less common, accounting for 1.4% of ectopic pregnancies.^3^ Blood supply is provided by the omentum or an intra-abdominal organ. Diagnosis by ultrasound is not easy and requires identifying a gestational sac outside the uterine cavity and adnexa.^2^ Cross-sectional imaging, particularly pelvic MRI, because of its low maternal-foetal risk, is often useful in confirming or adjusting the diagnosis. The use of MRI should not delay the management of patients for whom ultrasound has clearly diagnosed an EP. Instead, MRI should be reserved for situations where additional information is required to guide therapeutic decisions and cannot be obtained by ultrasound, and where patients are haemodynamically and clinically stable.^7^

MRI offers multiple advantages, including multiplanar imaging, the absence of ionizing radiation, superior soft tissue contrast to ultrasound and more specific characterization of tissues and fluids.^7^

The protocol involves T2-weighted images with single-shot fast spin-echo in all 3 planes, T1-weighted images without and with fat suppression to differentiate blood from fat, essentially in the axial plane, (coronal and sagittal are optional), as well as T2*-weighted images, which are useful for identifying haemorrhage and air bubbles. Furthermore, the use of gadolinium contrast agents is relatively contraindicated during pregnancy.^7^

In cases of heterotopic pregnancy, MRI shows a gestational sac outside the internal genital tract, located within the abdominal cavity with no myometrium surrounding it and with clear visibility of its own placenta. This location carries a higher risk of haemorrhage at the placental implantation site and increases the risk of maternal morbidity and mortality.^3^^,^^7^

Heterotopic pregnancy requires an early diagnosis and treatment in order to extract the ectopic sac while trying to preserve the IUP as much as possible. This approach also helps minimize the risk of future infertility and recurrence.^4^ It is most often performed surgically, by laparoscopy or laparotomy.^8^^,^^9^ When the diagnosis is uncertain, laparoscopic surgery may be required to confirm it without adding risk to the IUP through uterine manipulation. If the diagnosis is certain with no complications before 24 weeks of pregnancy, surgical removal is performed usually by laparoscopy.^3^ This method has been shown to reduce the risk of uterine manipulation and desiccation in comparison with laparotomy.^3^ Such risks associated with laparotomy include potential uterine irritability and postoperative spontaneous abortion.^10^ In cases of complicated pregnancy with hemodynamic unstability and haemoperitoneum, urgent surgical management is performed via laparotomy with salpingectomy for a ruptured tubal EP, salpingotomy, or oophorectomy. In certain difficult cases, a hysterectomy may also be required. Manipulation of the uterus must be kept to a minimum in order to preserve the IUP.^10^ Given the risk of severe bleeding from the placental attachment, it is recommended that the umbilical cord is ligated closed to the placenta, and the placenta is left in situ.^10^

Progesterone supplementation is sometimes necessary after surgical management of EP, when the IUP is progressive, in order to avoid post-operative abortion.^4^

Medical treatment may be considered in certain cases. For cornual EPs, this involves an ultrasound-guided potassium chloride injection.^10^ For non-cornual ectopic pregnancies where the ectopic sac is small, there is no internal bleeding, and the pregnancy is nonprogressive or nonviable, intramuscular methotrexate may be given as an alternative to surgical treatment due to its teratogenic effect and the higher abortion rate of the IUP in patients undergoing medical treatment as opposed to surgical intervention.^10^

## Conclusion

Heterotopic pregnancy represents a significant diagnostic challenge, due to the subtle clinical signs, elevated beta-hCG levels, and the visualization of an IUP, which can lead to a false sense of security, delaying diagnosis, and appropriate treatment. Pelvic ultrasound is the first-line imaging modality; however, an additional MRI may be required to confirm the diagnosis by visualizing a gestational sac outside the uterus, without surrounding myometrium, and clearly showing its own placenta. These imaging techniques allow for a conclusive diagnosis, guiding treatment decisions towards the most suitable approach.

This case illustrates the importance of a comprehensive understanding of clinical and radiological characteristics, as well as treatment options, that have made it possible to ensure the safety of both the mother and the normally developing twins in the uterine cavity.

## Learning points

Heterotopic pregnancy is a diagnostic and therapeutic emergency. Early diagnosis enables appropriate management, which essentially consists of surgical treatment.

Always check the adnexa, cervix, and entire abdominal cavity during the first-trimester ultrasound exam, especially if the woman has undergone MAP.

Ectopic pregnancy located in the abdomen can progress beyond the first trimester, increasing the risk of rupture of the ectopic sac, possibly threatening the mother’s prognosis.

Ultrasound is the modality of choice. An additional pelvic MRI can help confirm the diagnosis, showing a gestational sac in the abdominal cavity with its own placenta but without surrounding myometrium.
